# A Comparison of the Effect of Strength Training on Cycling Performance between Men and Women

**DOI:** 10.3390/jfmk6010029

**Published:** 2021-03-17

**Authors:** Olav Vikmoen, Bent R. Rønnestad

**Affiliations:** 1 Department of Physical Performance, Norwegian School of Sport Sciences, 0806 Oslo, Norway; 2 Section for Health and Exercise Physiology, Institute of Public Health and Sport Sciences, Inland Norway University of Applied Sciences, 2418 Elverum, Norway; bent.ronnestad@inn.no

**Keywords:** concurrent training, endurance performance, sex differences, muscle strength, muscle hypertrophy, training adaptations

## Abstract

During the last decade numerous review articles have been published on how concurrent strength and endurance training affect cycling performance. However, none of these have reviewed if there are any sex differences in the effects of concurrent training on cycling performance, and most research in this area has been performed with male cyclists. Thus, the aim of the current paper is to review the scientific literature on the effect of concurrent training on cycling performance in male and female cyclists with a special emphasis on potential sex differences. The results indicate that both male and female cyclists experience a similar beneficial effect from concurrent training on cycling performance and its physiological determinants compared to normal endurance training only. Some data indicate that women have a larger effect on cycling economy, but more studies are needed to explore this further. Furthermore, the adaptations to strength training thought to be responsible for the beneficial effects on cycling performance seem to be very similar between men and women. Interestingly, increased muscle cross-sectional area in the main locomotor muscles seems to be an important adaptation for improved performance, and, contrary to popular belief, cyclists should aim for increased muscle cross-sectional area when adding strength training to their normal training. We conclude that both male and female cyclists can improve their cycling performance by adding strength training to their normal training.

## 1. Introduction

Adding strength training to cyclists normal training has been shown to improve cycling performance [1,2,3], and during the last decade there has been published numerous review articles on the effects of strength training on cycling performance [4,5,6]. However, none of these have reviewed whether there are any sex differences regarding the effects of strength training on cycling performance. In fact, the literature investigating the effects of adding strength training to the usual training in cyclists is dominated by studies performed on male cyclists. One of the few studies investigating the effect of adding strength training to the usual training of female cyclists reported no beneficial effect on cycling performance in a 1 h time-trial test nor in cycling economy [7]. However, the cyclists in this study performed only one strength training exercise (parallel squats), and this was probably not a sufficient training volume for beneficial effects to occur [8]. A recent study reported improved average power output during and 8-min performance test in trained female cyclists after 6 weeks of either traditional or velocity-based strength training [9]. However, this study did not include a control group only performing endurance training, making it difficult to assess if the improved performance was because of the strength training per se. Furthermore, to the best of our knowledge, no study has directly compared the effects between men and women in one single study. There are some physiological differences between men and women that in theory might lead to different effects of strength training on cycling performance. For example, when performing muscle contractions at the same relative intensities, women have been reported to be more resistant to muscle fatigue [10], and during endurance exercise at submaximal intensities, women oxidize proportionally more fat and less carbohydrate and protein than men [11]. Furthermore, endocrine responses differ between men and women in response to exercise [12,13], and the adaptations in the mechanical properties of tendons after strength training are different [14]. Some years ago, we conducted a more thorough investigation on the effects of strength training on cycling performance and performance determinants in female cyclists [2,15]. This study utilized a strength training program, a testing regime, and testing equipment identical to what we used in male cyclists with beneficial effects on cycling performance [1,16,17], making these studies very suitable for comparing the effects between male and female cyclists. To the best of our knowledge, this study is the only study using a strength training program with a sufficient volume and training load to investigate if female cyclists can benefit from strength training.

In the current review, we first present a short summary of the effects of adding strength training to cyclists’ normal training regimes regarding cycling performance and performance determinants regardless of sex. Thereafter, we compare these effects between male and female cyclists with an emphasis on our own studies using a similar training program and testing protocol in both male and female cyclists. Finally, we include a review of the adaptations to strength training thought to induce beneficial effects on cycling performance and discuss if these might be different between men and women.

## 2. The Effects of Strength Training on Cycling Performance and Its Physiological Determinants

Cycling performance is determined by several physiological performance determinants. The interaction between maximal oxygen consumption (VO_2max_) and fractional utilization of VO_2max_ (%VO_2max_) determines the performance VO_2_, the rate of aerobic metabolism that can be sustained for the duration of a performance test or competition [18]. The cycling economy or efficiency then determines the power output at a given amount of energy consumption, and these three factors therefore majorly determine the average power output that can be sustained for a certain period of time or distance, a surrogate measure for cycling performance [18]. In the lab, cycling performance is often measured as the average maximal sustainable power output during 20–60 min tests.

Multiple studies reported improved cycling performance measured by this methodological approach after cyclists added heavy strength training (multiple leg exercises with ~4–12 repetitions maximum, for minimum 8 weeks) to their normal training [2,17,19,20]. In contrast, studies that included strength training programs of either short duration, included a low volume of strength training, or used explosive strength training [7,21,22,23] failed to show improved performance. Since combining heavy strength training with normal endurance training seems to improve cycling performance, it should also improve at least one of the cycling performance determinants. It seems to have neither a positive nor a negative effect on the development of VO_2max_ [2,7,22,24]. When it comes to efficiency or cycling economy, the findings are more equivocal. When cycling economy is measured by the traditional method (i.e., short, 3–5 min, submaximal bouts of cycling), no additive effect of strength training has been observed in well-trained and elite cyclists [1,3,17,25]. However, improvements have been shown in moderate trained cyclists [24,26,27]. Interestingly, there are indications that heavy strength training improves cycling economy after prolonged submaximal cycling, also in well-trained cyclists, which is especially relevant in road cycling [16].

%VO_2max_ is ideally measured directly via VO_2_ measurements during a performance test and then expressed as the average VO_2_ during the exercise in percent of VO_2max_. Only one study on the effects of strength training on cycling performance has measured %VO_2max_ in this way and reported an improvement after strength training [2]. However, a common way to estimate %VO_2max_ is to use the percentage of VO_2max_ at the lactate threshold, and the few studies that have reported the effect of concurrent training on this measurement in cyclists observed no change [2,17,24]. However, these data should be taken with caution as the only study reporting improved %VO_2max_ mentioned above did not find a similar effect of strength training on lactate threshold expressed as percent of VO_2max_ [2]. Furthermore, other studies report improved performance without a concomitant improvement in VO_2max_ or cycling economy [1,3] after concurrent training, further supporting the claim that improved %VO_2max_ can occur after concurrent training. The absolute power output at the lactate threshold is, amongst others, affected by the cycling economy. Therefore, and despite not all studies reporting significant improvement in cycling economy, the finding of improved lactate threshold power output in several studies after combined heavy strength- and endurance training is somewhat expected [1,2,17,20,25]. However, there are also studies observing no improvements in power output at a defined [la^-^] [3,23,24].

Another lab measurement that can be interpreted as a performance measurement is the peak minute power output achieved during an incremental cycling test to exhaustion when testing VO_2max_ (W_max_). W_max_ is influenced by VO_2max_, cycling economy, anaerobic capacity, and neuromuscular characteristics [28]. Accordingly, W_max_ has been shown to predict endurance performance in cyclists [29,30] and to distinguish elite cyclists from well-trained cyclists [30]. Concurrent endurance and heavy strength training is reported to increase W_max_ or time to exhaustion at W_max_ in trained to well-trained cyclists [1,17,19,24,31]. Another factor important for the cycling performance in mass start races is the ability to close a gap, break away from the pack, or perform well in the final sprint [32]. The outcome of these crucial moments is largely decided by anaerobic abilities and the size of the involved muscle mass [33,34]. Based on the beneficial effects of heavy strength training on muscle strength and muscle mass, it is as expected that concurrent training has been reported to improve the ability to generate a high power output for a short period of time [1,2,17].

## 3. Sex Difference in the Effect of Strength Training on Cycling Performance

**Figure 1:**
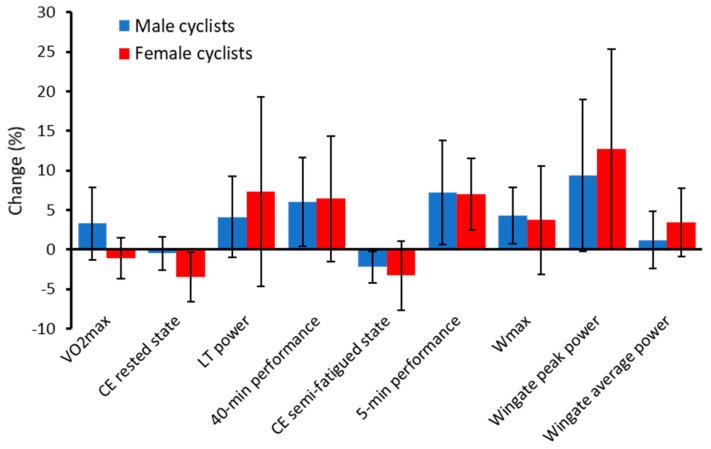
Percent change in physiological measurements and cycling performance after 11–12 weeks of heavy strength training in male and female cyclists. The results from the male cyclists are from [1,16], and the results from the female cyclists are from [2,15]. Values are mean ± SD. RM = repetition maximum, CSA = cross sectional area, VO_2max_ = maximal oxygen consumption, W_max_ = power output at VO_2max_, CE = cycling economy, LT = lactate threshold. Note: A negative change in cycling economy indicates reduced VO_2_ and improved cycling economy. CE semi fatigued state was measured during the last hour of a 3 h submaximal cycling trial, and the 5-min performance was measured directly following the 3 h trial. The increase in VO_2max_ in the male cyclists was not different from control male cyclists.

Since most studies investigating the effects of strength training on cycling performance include only male cyclists, we performed a study on the effects of strength training on cycling performance and performance determinants in female cyclists [2,15]. The female cyclists in this study performed a strength training program identical to what we previously used to induce beneficial effects on cycling performance in male cyclists [1,16,17]. This strength training program lasted for 11–12 weeks with two sessions per week with a training load of 10-4 RM, including four exercises for the lower body with three sets. Both the men and the women continued their normal endurance training. In both male and female cyclists, cycling performance, measured as the average sustainable power output during a 40-min performance test, increased to the same amount after adding strength training to their normal training (men: 6.0 ± 5.6%, women: 6.4 ± 7.9%, Figure 1). The improved performance was not because of increased VO_2max_ as strength training did not improve VO_2max_ compared to control cyclists performing their normal endurance training in either the male or female cyclists. Cycling economy measured by the traditional method (i.e., short, 3–5 min, submaximal bouts of cycling) was improved only in the female cyclists in our studies (Figure 1), and the percent change between men and women was significantly different (*p* = 0.016). Therefore, it is possible that female cyclists have a larger potential for improving cycling economy than male cyclists by adding heavy strength training to their normal endurance training. On the other hand, the male cyclists were on a somewhat higher performance level and completed more endurance training than the female cyclists (9.9 h vs. 5.1 h per week). It appears difficult to improve cycling economy in very well-trained cyclists [3]. In fact, improved cycling economy after heavy strength training has also been reported in male cyclists on a lower performance level [24,26,27]. However, research in very well-trained and elite female cyclists is lacking, and it might be that as for men, they do not see an improved cycling economy after strength training, at least when measured during 3–5 min submaximal bouts of cycling.

We measured %VO_2max_ with VO_2_ measurements during the 40-min performance test in the female cyclists and found an improvement from 78.9% to 82.2% of VO_2max_. Unfortunately, we did not perform this measurement in the male cyclists; however, the improved 40-min performance in the male cyclists compared to the male control cyclists with similar changes in VO_2max_ and cycling economy indicate an improved %VO_2max_. Similar findings have also been reported in other elite male cyclists [3]. This study found an 8% increase in the average power output during a 45-min performance test with no changes in cycling economy and VO_2max_ after young elite cyclists added strength training. The authors calculated that the average power output during the 45-min test increased from 76% to 83% of the power output corresponding to 100% of VO_2max_ after the strength training intervention. Therefore, it appears that both male and female cyclists improve %VO_2max_ after a period of heavy strength training. It might be speculated that the male cyclists increased their %VO_2max_ more than the female cyclists, since the improvement in 40-min performance test was similar despite only the female cyclists improved cycling economy (Figure 1). However, this might also be explained by the increase in VO_2max_ in the male cyclists (similar in both control and strength training group). It is therefore unknown if there are sex differences in the improvement of %VO_2max_ after concurrent training. The power output at lactate threshold improved similarly in both sexes after strength training (men: 4.1 ± 5.1%, women: 7.6 ± 12%, *p* = 0.39, Figure 1).

To simulate a typical mass start race in cycling, we performed a test consisting of a 3 h submaximal cycling trial (at the same absolute power output pre and post) directly followed by a 5-min performance test with the aim of highest possible power output during the 5-min test. The results were similar between the male and female cyclists [15,16]. Both had reduced oxygen consumption and hence improved cycling economy during the last part of the submaximal trial, compared to before the strength training intervention and the control groups. This was accompanied by reduced heart rate in both men and women. This shows that male cyclists on a high level can also improve cycling economy if this is tested in a semi-fatigued state. However, again there was an indication of larger potential for improvements in cycling economy after strength training in women than men, as they improved cycling economy during the last two hours of the test, compared to only the last hour in the male cyclists. The improved cycling economy during the last part of this prolonged trial probably led to a lower magnitude of fatigue at the end of the trial, and both the male and female cyclists increased average power output in the following 5-min performance test to the same degree after the strength training program (men: 7.2 ± 6.6%, women: 7.0 ± 4.5%, Figure 1). None of these changes were observed in either male or female control cyclists.

In our studies, we observed that strength training increased the ability to generate high power output for a short period of time, measured as peak power output in the Wingate test in both male (9.4 ± 9.6%) and female (12.7 ± 12.6%) cyclists. In both sexes, the effects on the 30-sec Wingate mean power were substantially smaller, with no significant increase in the male cyclists and only a small increase (3.4 ± 4.3%) in the female cyclists. The W_max_ also increased similarly in the male (4.3 ± 1.1%) and female (3.9 ± 6.7%) cyclists, although this change was only statistically significant in men.

The results from our studies show similar strength-training-induced improvements in cycling performance and physiological determinants of cycling performance between the sexes. Therefore, both male and female cyclists can incorporate heavy strength training into their training schedule for maximizing performance. However, the data indicates that the potential for improving cycling economy with strength training might be greater in women. Furthermore, caution is warranted since these studies did not compare men and women directly in the same study. However, similar strength training program, test protocols and testing equipment were used alongside a control group.

To the best of our knowledge, only one other study investigating the effect on concurrent training on cycling performance by comparing a concurrent strength and endurance training group to an endurance-training-only group has focused on female cyclists [7]. In this study, well-trained female cyclists added 12 weeks of heavy strength training (parallel squats two times per week) to their normal endurance training but found no beneficial effect on cycling performance in a 1-h time-trial test nor in cycling economy [7]. However, this might be because the strength training program only included one strength training exercise, making the total strength training volume too low for beneficial effects to occur [8].

## 4. Mechanisms behind the Effects of Strength Training on Cycling Performance and Sex Differences in These Mechanisms

**Figure 2:**
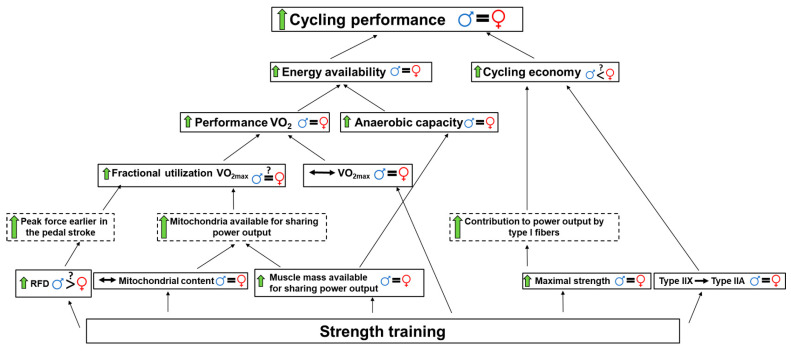
Flow diagram illustrating the proposed mechanisms behind improved cycling performance after strength training in cyclists with possible sex differences indicated with 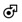 = men and 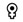 = women. Boxes with dashed line indicate theoretical effects of the strength training adaptation. = indicates similar change between sexes, > < indicates a larger improvement in one sex, question mark indicates uncertainty, green arrows represent improvement, 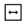 indicates no change, VO_2max_ = maximal oxygen consumption, RFD = rate of force development.

The proposed mechanisms responsible for improved cycling performance after strength training are summarized in Figure 2. One frequently proposed mechanism for the improved cycling economy often observed after cyclists add strength training to their normal training is a larger contribution of type I muscle fibers at a certain power output [4]. Type I fibers have been reported to be more efficient than type II fibers [35,36], and although not an universal finding [37], cycling efficiency has been related to proportions of type I fibers in the active muscles [38,39,40]. When the maximal muscle strength increases, the force levels required to ride at a certain power output is reduced relatively to maximal force. This implies that the type I muscle fibers can account for a larger proportion of a certain absolute power output [4,19], as follows from the size principle of motor unit recruitment [41]. Furthermore, concurrent heavy strength- and endurance training in female athletes has been reported to increase cross sectional area (CSA) of type I muscle fibers [42], and an increase in the force capacity and CSA of type I muscle fibers can theoretically induce a larger contribution to power output by the economical type I muscle fibers and/or postponing the activation of the less economical type II fibers [4]. The latter highlights the importance of increased muscle CSA in order to improve cycling economy through this mechanism. This is supported by the correlation (r = −0.54) that we reported between change in CSA of the quadriceps muscle and the improved cycling economy after strength training in the female cyclists [2].

Another often proposed mechanism is the increase in the proportion of type IIA muscle fibers at the expense of type IIX muscle fibers [2,4]. Some studies have reported that type IIA fibers are more economical than type IIX fibers in vitro [35,36,43,44]. Even though this difference in efficiency between type IIX and type IIA muscle fibers seems to be quite small at physiological temperatures [45], it could in theory improve work economy as well as improve endurance performance due to the larger endurance capacity in type IIA than IIX fibers [3,43]. The increases in muscle mass and muscle strength after strength training are reported to be very similar in men and women [46,47]. This is also the case for the transition from type IIX to type IIA after strength training that has been reported to be similar for both sexes in previously untrained subjects [48] and endurance trained athletes [2,3]. However, in our studies we observed a tendency toward a somewhat larger increase in quadriceps muscle CSA (women: 7.4 ± 5.3%, men: 4.6 ± 1.7%, *p* = 0.11) and lower body muscle strength (women: 38.6 ± 19.0%, men: 26.0 ± 6.6%, *p* = 0.08) in women than in the men that might explain the improved cycling economy in women and not in the men.

Increased tendon stiffness after strength training in men [49,50,51] is frequently proposed as an important mechanism behind improved running economy after concurrent training interventions [52,53,54], due to improved utilization of stored elastic energy and improved muscle contraction mechanics during the running stride. However, it has been observed that female tendons have a lower rate of new connective tissue formation and a lower mechanical strength in response to mechanical loading [55] with a different adaptation in mechanical properties of the tendons after strength training than men [14]. Therefore, it can be speculated that the strength training effect on tendon stiffness is smaller in women than men. However, cycling mainly consists of concentric muscle work [56,57] without a clear stretch-shortening cycle and any potential sex differences in tendon adaptations are likely to have no or only minor impact on cycling economy.

The mechanisms behind increased %VO_2max_ is unclear. However, it might be related to the increased CSA of the working muscles. The increase in quadriceps CSA in the female cyclists discussed above correlated with changes in the performance VO_2_ during the 40-min performance test (r = 0.59), which again is decided by %VO_2max_ and VO_2max_ [2]. %VO_2max_ is mainly determined by the amount of aerobic enzymes and mitochondria sharing a certain workload and VO_2_ [58,59,60]. It has been reported that cyclists that are able to spread the power output over a larger amount of their muscle mass has a larger %VO_2max_ during a 60-min time trial test [61]. Therefore, we have previously suggested that one possible explanation behind improved %VO_2max_ after strength training can be that increased muscle CSA leads to more muscle mass being available to share a certain power output. Since most longitudinal training studies report unchanged content or activity of aerobic enzyme in previously untrained individuals after strength training [62,63,64], the total amount aerobic enzymes (mitochondria) available for sharing the workload should be increased [2]. In the female cyclists, we reported no change in the concentration of aerobic enzymes despite an increase in muscle fiber CSA and total quadriceps muscle CSA giving support for this mechanism [2,42].

A frequently proposed mechanism for improved endurance performance after heavy strength training is an increase in rate of force development (RFD) [3,24,65]. Increased RFD may reduce the time to reach the force needed to sustain a certain power output and may thus allow for a prolonged relaxation phase in each pedal stroke, and consequently facilitate better blood flow to exercising muscles [3,4]. If increased RFD improves performance, thereby allowing cycling at a higher mean power output, this will increase oxygen demand and therefore also performance VO_2_. If VO_2max_ remains unchanged, this will lead to increased %VO_2max_. Even though concurrent strength and endurance training can reduce the improvement in RFD compared to strength training alone [66,67], increased RFD and earlier peak force during the pedal stroke are likely contributors to improved %VO_2max_ after strength training. A study from our group demonstrated that elite cyclists exhibited earlier peak torque in the pedal stroke following a strength training program like the one used in our other studies discussed above. Furthermore, this earlier peak torque correlated (r = −0.63) with a concomitant improvement in mean power output during a 40-min performance test [17]. As the intended movement velocity is important for changes in RFD when training with high loads [68] cyclists should perform strength training with maximal intended movement velocity for maximizing improvements in RFD.

As most studies report similar relative improvements in muscle mass or muscle CSA between men and women, any increase in %VO_2max_ through an increased muscle mass available for sharing a certain power output should be similar between the sexes. Changes in RFD in absolute values after strength training are mainly because of increased maximal strength, increased muscle mass, and changes in rapid activation of the muscles, and these adaptations are similar between sexes [47]. Increased stiffness of tendons might theoretically also contribute to increased RFD as stiffer tendons will transfer the force from the muscles to the bones faster [47]. Therefore, it might be speculated that men would have larger increase in RFD since they appear to have larger increase in tendon stiffness. A recent meta-analysis indicated that men increase RFD to a larger degree than women after strength training, but these results were unclear because of shortage of data from female subjects [68]. Therefore, the adaptations to strength training that probably lead to improved %VO_2max_ in cyclists seem to mostly be similar for men and women, further supporting that strength training will improve %VO_2max_ in both men and women. However, future studies should further investigate if there might be sex differences in the magnitude of this improvement.

As rationalized, a small increase in CSA in the main locomotor muscle seems to be an important factor, both for the improvement in cycling economy and %VO_2max_ and hence cycling performance after strength training. In our study with the female cyclists, the increase in muscle CSA correlated with both improved cycling economy (r = −0.54), improved performance VO_2_ (r = 0.59), and improved 40-min performance (r = 0.73) [2]. Furthermore, increased muscle CSA is also important for the ability to generate high power output for a short period and anaerobic abilities [33,34]. Based on these findings, female and male cyclists that add strength training to their normal training should aim for increased muscle CSA in the important muscles for power output when cycling. This contrasts with popular belief among cyclists and coaches, who usually try to avoid increased muscle CSA in fear of negative consequences of increased body mass. However, it is important to remember that a small increase in muscle CSA in the main locomotor musculature does not have large impact on total body mass. In fact, in our studies we do not report increased body mass despite significant increases in quadriceps muscle CSA, and performance is also improved when adjusted for body mass in both female [2] and male cyclists [1]. Furthermore, the increased muscle CSA does not impair capillary density in either female or male cyclists [2,3]. Based on the limited available literature in female cyclists, it is difficult to say if the optimal strength training program to improve cycling performance should be different between male and female cyclists. In our studies, the strength training program was identical and produced a similar improvement in performance. There is also limited evidence indicating that the mechanisms behind improved performance are different. Therefore, there are no indications that male and female cyclists should use different strength training programs, but this should be explored in future studies.

## 5. Summary

Our review of the literature, which focuses on our own studies using similar strength training and test measurements in male and female cyclists, shows that both sexes improve cycling performance and therefore can add heavy strength training to their normal training in order to improve performance. The improved performance seems to be because of both improved cycling economy and %VO_2max_. Both moderately trained male and female cyclists have been reported to improve cycling economy after heavy strength training. However, it seems to be difficult to improve cycling economy after concurrent training in very well-trained and elite male cyclists, indicating that improvement in cycling economy is easier in women. However, studies including elite female cyclists are lacking, and there are indices of improved cycling economy even in well-trained male cyclists when tested after prolonged cycling in semi-fatigued state. The improved %VO_2max_ seems to be similar between male and female cyclists after concurrent training. However, future studies should include direct measurements of %VO_2max_ with sufficient numbers of both male and female cyclists with similar training background that in parallel carries out the same strength training program to directly compare the effects between sexes and further explore if there might be differences. The physiological adaptations after strength training believed to be responsible for these improvements seem mostly similar between sexes. Improvements in the ability to generate high power output for a short period of time and anaerobic abilities after strength training also seem to be similar between male and female cyclists.

## Data Availability

Not applicable.
